# Designing and Implementing an Innovative SMS-based alert system (RapidSMS-MCH) to monitor pregnancy and reduce maternal and child deaths in Rwanda

**Published:** 2012-10-14

**Authors:** Fidele Ngabo, Judith Nguimfack, Friday Nwaigwe, Catherine Mugeni, Denis Muhoza, David R Wilson, John Kalach, Richard Gakuba, Corrine Karema, Agnes Binagwaho

**Affiliations:** 1 Ministry of Health, Rwanda; 2 UNICEF Rwanda; 3 Musanze District Hospital, Rwanda; 4 Management Sciences for Health, Rwanda; 5 Department of Social Medicine, Harvard Medical School, Boston, USA

**Keywords:** mHealth, eHealth, SMS, RapidSMS, Maternal and Child Health

## Abstract

**Introduction:**

With the continuous growth of mobile network coverage and unprecedented penetration of mobile devices in the developing world, several mHealth initiatives are being implemented in developing countries. This paper aims to describe requirements for designing and implementing a mobile phone-based communication system aiming at monitoring pregnancy and reducing bottlenecks in communication associated with maternal and newborn deaths; and document challenges and lessons learned.

**Methods:**

An SMS-based system was developed to improve maternal and child health (MCH) using RapidSMS^®^, a free and open-sourced software development framework. To achieve the expected results, the RapidSMS-MCH system was customized to allow interactive communication between a community health worker (CHW)following mother-infant pairs in their community, a national centralized database, the health facility and in case of an emergency alert, the ambulance driver. The RapidSMS-MCH system was piloted in Musanze district, Nothern province of Rwanda over a 12-month period.

**Results:**

A total of 432 CHW were trained and equipped with mobile phones. A total of 35,734 SMS were sent by 432 CHW from May 2010 to April 2011. A total of 11,502 pregnancies were monitored. A total of 362 SMS alerts for urgent and life threatening events were registered. We registered a 27% increase in facility based delivery from 72% twelve months before to 92% at the end of the twelve months pilot phase. Major challenges were telephone maintenance and replacement. Disctrict heath team capacity to manage and supervise the system was strengthened by the end of pilot phase. Highly committed CHWs and effective coordination by the District health team were critical enablers.

**Conclusion:**

We successully designed and implemented a mobile phone SMS-based system to track pregnancy and maternal and child outcomes in limited resources setting. Implementation of mobile-phone systems at community level could contribute to improving emergency obstetric and neonatal care, yet it requires a well-organized community health structure in limited resource settings.

## Introduction

Progress in reducing maternal mortality in developing countries has been rather slow to meet the Millennium Development Goal (MDG) target number 5, aiming to improve Maternal Health by reducing maternal mortality by 75% between 1990 and 2015 and achieving universal access to reproductive health by 2015 [1]. Developing countries account for 99% of maternal death worldwide, representing one of the widest health gaps between developed and developing countries. These deaths could be avoided if the proper health resources and services were made available to women. Many factors are contributing to this including low level of education, knowledge and economic power of women to make informed reproductive health decisions; limited scale or lack of effective sexual and reproductive health programmes [2]. In most developing countries, the underlying determinants of maternal death during pregnancy and postpartum are affected by the three-delay from (i) identifying a life threatening event and making the decision to go to the health facility, (ii) reaching the health facility, and (iii) intervening effectively [3].

The use of mobile and wireless devices to support medical and public health practice and research (mHealth) is gaining increased attention as it provides opportunities to rapidly connect people, reducing therefore delay across the chain of health decisions, and positively affecting the lives of millions of underserved population [4]. The International Telecommunication Union (ITU) estimated at 5.9 billion the number of mobile subscribers in 2011, with global penetration estimated at 87% of which 79% in the developing world [5]. With the continuous growth of mobile network coverage and unprecedented penetration of mobile devices in the developing world, several mHealth initiatives are being implemented in developing countries.

Various mHealth initiatives have been piloted in sub-Saharan African including Uganda's RapidSMS system to support the management of the roll out of malaria's rapid diagnostic test by the country's National Malaria Control Program [6]; Zambia's SMS system to reduce delay in sending infant HIV testing results from centralized laboratory to remote rural health facilities [7], and Malawi's SMS system to improve communication among health workers for family planning and reproductive health in rural areas [8].

Rwanda is the most densely populated country in Africa with 415 inhabitants per square kilometre. The newly released Rwanda Demographic Health Survey (DHS) estimates Maternal Mortality Ratio (MMR) at 476 per 100,000 live births [9] down from 750 per 100,000 estimated in 2005 [10]. Nevertheless, Rwanda is among the sub-Saharan Africa countries where the largest total percentage decline of MMR (51% reduction) was registered between 1990-2008 [1]. Despite the above stated progress, the country has yet to meet the MDG 5, corresponding to 325 per 100,000 live births. Rwanda has recently developed a comprehensive Information Technology strategy plan that includes eHealth (the combined use of electronic communication and information technology in the health sector) and mHealth as significant components.

This paper describes the design and implementation of a mobile phone-based communication system aiming at monitoring pregnancy and reducing the three delays in communication associated with maternal and newborn deaths in Rwanda, and documents challenges and lessons learned.

## Methods

This section describes the context and approach applied in implementing RapidSMS-MCH system.

### Structure of the health system in Rwanda

Rwanda adopted a health development strategy based on decentralized management and district-level care from the 1980s [9]. At central level, The Ministry of Health (MOH) and its units provide the strategic vision and stewardship for national programs, setting of norms and standards, and the monitoring of central/referral hospitals. At District level, the district health office is responsible for the planning management, coordination and evaluation of health service delivery at district level down to sector, cell and village level [11]. From 2004, Rwanda adopted and gradually scaled up a national system for Performance Based Financing (PBF) to promote good clinical practice and quality outcomes [12]. The current health system model was designed to cater for the primary preventive needs of communities in particular and primary curative needs in general.

### Community Health Program in Rwanda

The Rwanda Community health program was established in 1995, aiming at increasing uptake of essential maternal and child clinical services through pregnant women education, promotion of healthy behaviour, and follow-up and linkages to services. The first line of service delivery is provided by over 45, 000 Community Health Workers (CHW) operating at village level and including: (1) the “binomes” (a male-female CHW pair) providing basic care; (2) CHW in charge of Maternal Health. The CHWs are not formal health professionals, but rather volunteers elected by community members. The CHWs receive training and supervision through the health system. The Three aforementioned CHWs operate in each village with clearly defined roles and responsibilities. Throughout this paper all mention of CHW will be referring to CHWs in charge of maternal health. The CHW activities in the community consist of identifying pregnant women, making regular follow-ups during and after pregnancy and ensuring deliveries in health facilities and by skilled health worker.

### RapidSMS-MCH system's objectives

The RapidSMS-MCH system was designed to provide an SMS-based platform, enabling effective and real-time two-way communication for action, between CHWs at community level, and the rest of the health system (ambulance, health facility staff, District Hospital and Central level) through mobile phones. The primary expected result of the system is an improved access to antenatal, postnatal care, institutional delivery, and emergency obstetric care. In addition, RapidSMS provides a database for keeping clinical records of maternal care delivery.

### Implementation site

This pilot phase was implemented in Musanze, Rwanda's most mountainous district located approximately 85 Km (53 miles) north of Kigali the capital. Musanze's population is estimated at 347,692. The percentage of assisted deliveries in Health Facilities was estimated at 48.84% in 2009 [13] ranking among the lowest in the country. All the above characteristics made Musanze the ideal site to pilot the project. The district comprises 13 health centres and one district hospital. Human resources include 18 General Practitioners, 5 Specialists and 300 Nurses. Additionally, the district benefit from services of 1,296 CHWs, including 432 CHWs specifically tasked with maternal and new born health. Each health centre has a community health officer (supervisor) supervising an average of 55 CHWs operating in the villages within the health facility's catchment area. A community health representative working at district hospital level manages the supervisors from the facilities. This same structure is common to all districts in the country. The expected number of pregnancies in Musanze was about 14,200 per annum, giving an average of 30 pregnancies per year and 2-3 per month to be identified and managed by each CHW. This was viewed as a reasonable level of work.

### RapidSMS-MCH system design, development and implementation

Using the RapidSMS platform - an innovative SMS-based technology developed by UNICEF, the Rwanda Ministry of Health designed a RapidSMS-MCH system, customized to establish a communication and alert system, support documentation of pregnancies in the community, increase health facility contact through antenatal care, institutional delivery and hence by proxy, professional care at birth.

**Figure 1:**
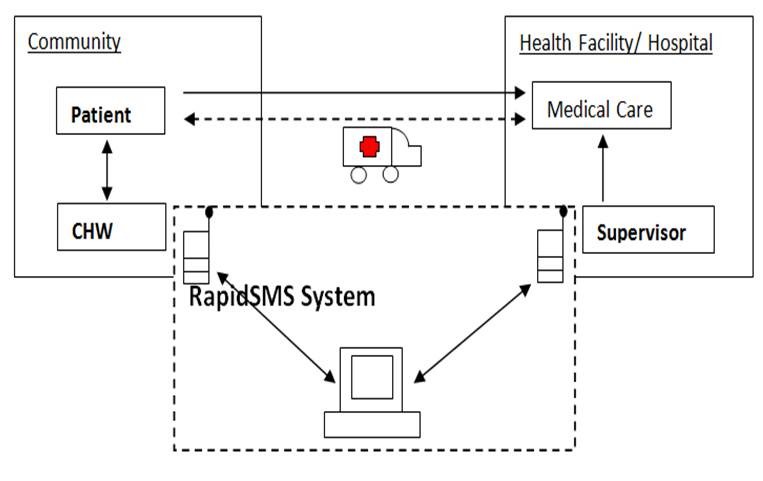
Maternal health care configuration at sector level. Former configuration consisted of patient and CHWs in the communities on one side and the facility on the other side. Emergency obstetric care was lacking especially for those patient located at a long distance from hospital. The newly implemented RapidSMS-MCH system offers a linkage between the patient in the community and the appropriate level of care.

RapidSMS-MCH system offers a tool to CHW for registration of new pregnancies in their respective community and effective monitoring throughout the pregnancy up to delivery and post-partum. Each community has an elected CHW in charge of Maternal and Child health. They sensitize community members about needs to attend antenatal care, and provide support and referral to the health centre for antenatal care, particularly in case of life threatening event (danger signs). CHWs register new pregnancies in the system, and report all danger signs. For a normal pregnancy, the RapidSMS-MCH system will send automated reminders at specific date for clinical appointments, including delivery. In case of danger signs, an emergency alert-system is triggered, and provides immediate feedback to the CHW, advising on immediate action. Ambulance requests are forwarded to the nearest ambulance vehicle point to ensure that mother/infant is timely transferred for emergency obstetric and neonatal care (Figure 1).

### RapidSMS-MCH system application design

RapidSMS ^®^ is an open source SMS application platform written in Python and Django [14, 15]. The SMS-based project was developed to track the pregnancy lifecycle; enabling instant reporting of pregnancy related event and timely notification for emergencies, alerting health facilities, hospital and ambulances. We developed the system requirements to meet those expectations. Between February and March 2010, the programmers developed the first version of the SMS-based application which was temporally running on a simple desktop computer linked to a modem with a Subscriber Identity Module (SIM) card and short code number obtained from Rwanda's Utility Regulatory Agency. The SIM card provided by the mobile operator MTN Rwanda was reverse billing enabled thus allowing end-users to send SMS without any incurred charge. The Rwandan Ministry of Health covered SMS cost within the framework of this project.

We used an iterative development approach [16] relying on feedback from Musanze District hospital and MOH staff and from regular field testing. The feedbacks were analysed, documented and used to improve system functionalities. The application was later moved to a server and connected to a mobile gateway with enhanced capability to handle multiple and simultaneous SMS queries from the system. The final version was installed in a central server and linked to a public IP address.

### SMS form design

Eight SMS forms were designed to capture information related to specific maternal and child health events (Table 1). We designed each SMS to start with a special keyword followed by several fields within the 160 characters limit.

### Description and functionalities of the system

**Figure 2:**
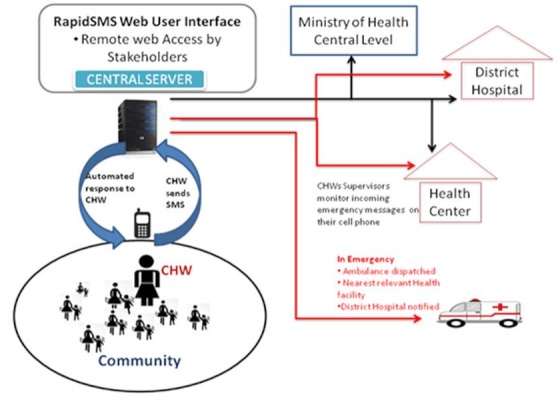
RapidSMS-MCH system information flow

Figure 2 presents the system's workflow in the RapidSMS-MCH system. The system allows a two-way flow of information. A registered CHW creates and send an SMS to the system using a short code telephone number. The message received by the server will immediately trigger a specific feedback to the sender. For each registered pregnancy, the system will send automated reminders of forthcoming antenatal care visit and due date of delivery to the CHWs. But it is important to note that the reminder SMS is sent to the CHW's phone and not the Patient's phone.

In case of danger signs (haemorrhage, in labour and at home, and unknown serious condition) during pregnancy and reported to the CHW by a pregnant woman at village level, the CHW sends an emergency SMS alert to the system. Consequently a message is generated and sent simultaneously to the driver of nearest ambulance vehicle and the manager of facility for immediate intervention. This SMS includes the danger sign reported, the name of the village and telephone number of health worker who sent the original message. Another SMS is sent to the CHW indicating immediate action to manage the danger sign and prepare pending ambulance arrival.

The system incorporates features enabling continuous technical monitoring to recognize and record, in an error-log, inconsistencies such as wrong SMS formatting or logic mistakes indicating that a reporter may be having difficulties with reporting. Upon reception of such messages, the system replies to the reporter with suggestions of how to revise en resend the message with the correct format. The error log contributes to facilitate supervision of CHWs. It is a very useful source of information for the supervisors who use them to provide feedback to the CHWs.

### Web User interface

**Figure 3:**
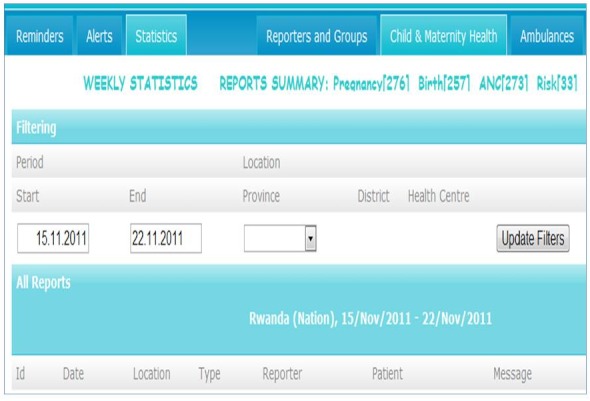
Web user interface

A password protected Web user interface gives access to aggregated and disaggregated data and enables individual patient history tracking as well as output of reports. The password protected web user interface presents an overview of the system's outputs including individual and aggregated reports, statistics, and system administration, log of reminders and activity of CHWs (Figure 3). Data can be filtered at three levels: national, district and health facility level. A user working in a health facility at district level will be assigned an account, granting him/her permission to view and possibly modify data only from his health facility's catchment area.

### Training of Community Health Workers

A cascade training approach was adopted, consisting of an initial training of ten national trainers affiliated with the MOH and partners working in the district (UNICEF, MSH). National trainers were encouraged to take part in the development of training material. Second stage of training consisted of one-day training of 24 Community Health supervisors and data managers, operating at district level and conducted by the national trainers. The third and last stage of training consisted of two-day training of 432 CHWs by the 24 supervisors and data managers. 500 Telephones were distributed to the CHWs in charge of Maternal and Child Health and health facilities in Musanze district, marking the official launch of the RapidSMS-MCH system. Training materials were translated in local language (Kinyarwanda). Each training session of the CHWs was conducted by two trainers and included a maximum of 20 trainees. Training was followed by intensive follow up supervision, and refresher training and feedback sessions to ensure effective capacity transfer. Performances of CHWs were regularly assessed through analysis of data sent into the system and error logs.

## Results

**Figure 4:**
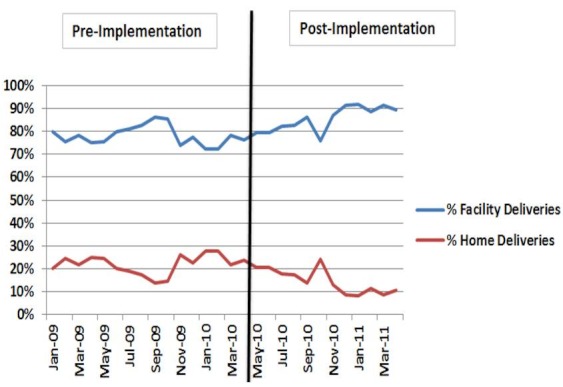
Trend in facility and home deliveries in Musanze District, January 2009 - May 2011

Between May 2010 and April 2011, 35,734 reports were submitted in the system, including 11,502 pregnancies registrations (81% of the 14,200 estimated annual pregnancies in the district). 3 maternal deaths and 137 child deaths were registered in the system. SMS reminders were sent out to the CHW's phone in relation to each registered pregnancy. Reporting compliance among CHWs was 100%. The percentage of delivery in health facility and at home varied respectively between 72 and 92% and between 8 and 28% (Figure 4).

CHWs reported being more pro-active in finding new pregnant women and following up registered pregnant women as a result of reminders forwarded to their mobile phones. Error rate (the number of SMS sent with formatting errors) dropped from 54% in the first four months to 8% by the end of April 2011.

The number of SMS-alert associated with danger signs was 163. The most common danger sign reported was antepartum and postpartum hemorrhage, accounting for 30% of emergency cases.

## Discussion

Rwanda designed and implemented a mobile phone-based system in Musanze district between May 2010 and April 2011 with the aim of monitoring pregnancy and reducing bottlenecks in communication associated with maternal and newborn deaths. Usage patterns and challenges encountered by end users were collated and used to make adjustments to the overall system (SMS form, system's application, etc.). Early results of this initiative prompted Rwanda MOH to develop a national scale-up plan. At the time this paper was published the project had rolled out to 18 out of the 30 districts nationwide. A total of 15,000 phones were distributed to more than 7000 CHWs were trained.

Locally based software development expertise was used in the present project from a strong recommendation made by MOH and consisting of using and building local expertise to promote sustainability of the program. Consequently an International expert in open source SMS application development was consulted for three weeks to train two local programmers recruited to develop the RapidSMS-MCH application. The downside of relying on programmers with limited experience appeared to be an initial slower pace experienced with regard to system deployment.

This is likely one of the first large scope mHealth projects having gone beyond pilot phase and addressing bottlenecks associated with increased maternal and child mortality in resource-limited settings. The project helped to reduce the delay in seeking health care through a short SMS sent by a community health worker alerting the health system for timely and appropriate medical assistance. The system can also serve as the most remote data entry point for pregnant women and children health status into the national health information systems, and as unique interface to provide real-time data to health facilities staff, and program manager.

### Limitations

The lack of redundancy in the system was a limitation in the initial system design. The system was designed to forward ambulance requests from communities to the health centres and to an ambulance driver. However, considering a situation where the ambulance is out of network or out of service for a reason beyond human′s control, the system could potentially fail to do what is was intended which is act as an alert system aiming to trigger emergency and obstetric care without delay. A solution to this failure was to forward the ambulance requests to at least one additional individual at the district health office. This was automated in the system prior to scale-up.

Another limitation of the present project was the inability of the system to generate two important statistics. In fact, it was not possible to disaggregate statistics of the numbers of registered pregnant women by number of attended antenatal care visits. That system's failure, which has now been adjusted, was due to an error found in the initial structure of the RapidSMS-MCH application. Additionally, neonatal deaths were not differentiated from other infants’ deaths, making it impossible to establish the number of new born deaths registered during the pilot phase of this project. This limitation was found during data analysis and quality assurance. However, feedback sessions with CHWs were used to highlight and address the gaps found in SMS reporting.

This paper is not an evaluation of a mobile phone-based system to decrease maternal death. Statistics presented in the result section are merely presenting, on the one hand, the trends observed in MCH care after initiation of mHealth and, on the other hand the potentials offered by mHealth in low resource settings.

### Lessons Learned and Challenges

Favourable programming factors in the Rwanda context included an already existing and well organized community based health program, the PBF approach coupled with the scale-up of community health insurance, and good cell phone coverage reaching even the most remote areas of the country. Furthermore, the perfect delineation of administrative boundaries with clearly defined roles and responsibilities for CHWs facilitated monitoring and quality assurance. The CHWs were instrumental in addressing the infrastructural and geographical barrier that negatively impact access to care in most low resources countries. In Rwanda, CHWs are organized in cooperatives in each sector of the district, and their income is mostly based on performance (PBF program). The most critical enabler in Rwanda was the strong Government commitment to innovation in general and the RapidSMS-MCH initiative from inception. This was also mentioned by Jim Barrington and others regarding the SMS pilot project to improve anti-malarial drug supply management in rural Tanzania [17].

Given the scope of the project, initial investment cost in setting up the SMS-based system was considerable. That was due to the fact that MOH had made the commitment to provide mobile phones to every CHW in the entire country. Other authors have discussed using CWH's personal phones to minimize initial investment [6, 17]. Rwanda's choice to provide the phones was perceived as a guarantee to boost engagement and motivation of CHWs from the start.

Furthermore, MOH engaged the private sector in a public-private partnership to substantially lower recurrent cost of SMS. As a result, the initially agreed SMS cost dropped by 10 times from 30 Rwandan Francs (0.05 USD) to 3 Rwandan Francs (0.005 USD). The communication cost reduction was crucial in planning for expansion and ensuring sustainability.

A slight increase of facility deliveries was noted at the end of pilot of the project. But, that was merely an observation without strong causality and should bring about the need to apply a more comprehensive evaluation to determine the effect of this SMS-based system intervention on facility-based delivery.

An unexpected benefit of RapidSMS-MCH system was the ability to assess CHW's performance through the web-interface dashboard [18]. The RapidSMS-MCH system proved to be a powerful tool for CHW supervision necessary for successful community health programs. Previously it was challenging for the supervisors of CHWs to carry out supervision given the distance and lack of transportation means. The system's web-user interface giving access to CHW's activity reports helped mitigate the distance issue, and allowed supervisors to track and monitor the productivity of CHWs.

Telephone maintenance was a common challenge reported in addition to lack or limited access to electricity in a number of communities. The CHWs were recommended to recharge their telephones at the closest health centre. But, the CHWs positioned far from health centres were challenged by long walking distance on a regular basis. CHWs in the present project admitted befitting of more trust and respect in their communities as a result of being empowered to request an ambulance in case of emergencies. The RapidSMS-MCH system has therefore contributed to put a mobile phone, a powerful tool, into the hand the female CHWs in Musanze district. This has been observed in other studies by GSMA and others studying opportunities offer to women through mobile phone in low and middle-income countries [19].

It is worth noting that an assessment of the RapidSMS-MCH system perception among CHWs revealed how intrigued and delighted the project beneficiaries (pregnant women) felt to be reminded of their next ANC visit or estimated delivery date. In fact, because only clinically confirmed pregnancies were registered in the system, women were incited to attend antenatal care in order to have their information recorded in the central SMS database and subsequently take advantage of the reminders and ambulance alert service.

## Conclusion

RapidSMS-MCH demonstrates that mobile phone offers an opportunity to overcome barriers that limit access to quality maternal and child health. Mobile phones can help women, their families, and local health workers to seek timely, appropriate medical help for an obstetric and new born emergency by reducing the time that elapses between a health crisis and care. However, its replication and sustainability will depend on country specific community health program context, a strong government and private sector commitment.

## Additional material

**Additional material:** SMS forms used for sending report to the central server (PDF 45kb)
